# Improved chlorination and rapid water quality assessment in response to an outbreak of acute watery diarrhea in Somali Region, Ethiopia

**DOI:** 10.2166/washdev.2020.146

**Published:** 2020-06-24

**Authors:** Anu Rajasingham, Ben Harvey, Yodit Taye, Stanislaus Kamwaga, Andrea Martinsen, Mohamed Sirad, Mowlid Aden, Kathleen Gallagher, Thomas Handzel

**Affiliations:** Emergency Response and Recovery Branch, Division of Global Health Protection, Centers for Disease Control and Prevention, Atlanta, USA; Ethiopia WASH Cluster, Addis Ababa, Ethiopia; Somali Regional Water Bureau, Jijiga, Ethiopia; Somali Region WASH Cluster, Jijiga, Ethiopia; Emergency Response and Recovery Branch, Division of Global Health Protection, Centers for Disease Control and Prevention, Atlanta, USA; Somali Regional Water Bureau, Jijiga, Ethiopia; Somali Region WASH Cluster, Jijiga, Ethiopia; Ethiopia Country Office, Centers for Disease Control and Prevention, Addis Ababa, Ethiopia; Emergency Response and Recovery Branch, Division of Global Health Protection, Centers for Disease Control and Prevention, Atlanta, USA

**Keywords:** acute watery diarrhea, centralized chlorination, chlorination, rapid assessment, water quality, water treatment

## Abstract

Somali Region of Ethiopia has been affected by drought for several years. Drought conditions have led to food and water scarcity and a humanitarian crisis in the region. In January 2017, an outbreak of acute watery diarrhea (AWD) was declared in the region. AWD prevention and control activities include strengthening water, sanitation, and hygiene (WASH) services. Access to safe drinking water is critical in preventing transmission of AWD and chlorine is an effective chemical to disinfect water supplies. The US Centers for Disease Control and Prevention collaborated with the WASH Cluster and the United Nations Children’s Fund, Ethiopia, to provide technical assistance to the Somali Regional Water Bureau to improve chlorination of drinking water supplies and quickly assess water quality improvements in Jijiga town, Fafan Zone. Timely sharing of surveillance and case investigation data allowed for the identification of gaps within the water supply system in Jijiga and implementation of centralized and decentralized chlorination interventions and monitoring systems. Pilot use of a rapid assessment to determine residual chlorine levels at various points in the city helped improve chlorination intervention impact. This work illustrates that rapid community-level water quality improvements can be implemented and assessed quickly to improve interventions during outbreaks.

## BACKGROUND

Acute watery diarrhea (AWD) prevention and control activities include strengthening water, sanitation, and hygiene (WASH) by ensuring access to safe water supplies, treatment of drinking water at the community or household level, provision of sanitation facilities, and promotion of handwashing and food safety (Taylor et al. 2015). Access to safe drinking water is critical in preventing transmission of AWD and chlorine is an effective chemical to disinfect water supplies and protect water from recontamination during handling and storage. During outbreaks and emergencies, the free residual chlorine (FRC) level of drinking water is recommended to be 0.2–0.5 mg/L at the point-of-use, 1.0 mg/L at tapstands, and 2.0 mg/L at water trucks during filling (WHO 1993; MSF 2010). Routine monitoring of FRC levels is important to ensure the continued provision of safe water (WHO 2016).

Drought has affected the Somali Region of Ethiopia since 2015. The resultant impact on crop and livestock cultivation has led to food and water scarcity, population displacement, and a humanitarian crisis in the region. This crisis was further exacerbated by an outbreak of AWD that started in January 2017. From 1 January to 10 July 2017, there were a total of 33,993 cases in the region including over 1,000 cases in Jijiga, the capital of the region.

Jijiga is the largest urban area in Somali Region and a transportation hub, with population movement in and out of the region and country. Thus, improving access to safe water in Jijiga was considered critical to responding to the outbreak in the region. A variety of water sources are used to provide drinking water to the approximately 159,300 residents of Jijiga. Water supplied by the piped system is sourced from deep boreholes and stored in two reservoirs 1,000–3,000 cubic meters in volume before release. The Somali Regional Water Bureau (RWB) estimates that the piped network reaches 80% of residents in Jijiga primarily through communal tapstands. However, piped water is only available for several hours a day resulting in queuing at tapstands. In addition, the piped system supplied by the reservoirs does not reach all areas of Jijiga. The remainder of residents collect water from a variety of sources, including donkey carts, water trucks, and local boreholes, not connected to the piped network. The water sources for the donkey carts and water trucks vary. Some collect directly from the piped network, others from filling stations, and still others fill with surface water from a nearby dam. Initial FRC testing conducted in Jijiga before the intervention determined that water supplied from the reservoirs was not consistently chlorinated and that water from donkey cart filling stations and truck filling stations was not chlorinated.

We identified two main challenges that compromised the drinking water quality in Jijiga. First, although water supplied by the reservoirs was chlorinated at times, no consistent treatment and monitoring plans were in place. Second, donkey carts and trucks that filled from filling stations were not chlorinated and those that were filled from the piped network did not receive booster chlorination doses, despite longer transport times and infrequent tank flushing. To address these challenges, the US Centers for Disease Control and Prevention (CDC), in collaboration with the Somali Region WASH Cluster and the United Nations Children’s Fund, Ethiopia (UNICEF), provided technical assistance to the Somali RWB to improve chlorination of drinking water supplies in Jijiga and water quality monitoring. Our report details the chlorination interventions implemented and focuses on the use of a rapid assessment exercise to quickly collect data to improve intervention impact.

## METHODS

The CDC, WASH Cluster, and UNICEF supported the Somali RWB in several areas. First, partners identified and mapped priority water points in AWD affected areas of Jijiga by using AWD surveillance and case investigation data. Second, appropriate chlorination methods were implemented at water points. Centralized chlorination was used to improve chlorination through the piped network, and trucks and donkey carts were chlorinated at filling stations using decentralized chlorination methods - bulk chlorination. Third, the CDC and the Somali RWB trained all attendants in charge of releasing water at the city’s reservoirs, truck, and donkey cart filling stations on chlorination procedures. Attendants at the reservoirs were trained in how to chlorinate daily during reservoir filling using high-test hypochlorite (HTH) with a target dose of approximately 1.0 mg/L. Filling station attendants at truck and donkey cart filling stations were trained to dose trucks and carts at 2.0 mg/L. Fourth, to supplement the chlorination efforts, a water quality monitoring phone application was created for RWB in collaboration with UNICEF, CDC, and IRC to monitor FRC levels at different water points serving the community, including tapstands, truck filling stations, and donkey cart filling stations. The application was designed so that water quality data could be entered over time and tracked by location. Fifth, a Somali RWB staff member was trained to conduct regular monitoring visits to the reservoirs and filling stations to ensure chlorination efforts continued. Lastly, in partnership with the WASH Cluster, a free residual rapid assessment was conducted to quickly assess chlorination interventions. The implementation and findings of the rapid assessment are the focus of this short communication.

On 15–16 June, approximately 1 month after the initiation of chlorination activities in Jijiga, the WASH Cluster conducted an FRC rapid assessment to evaluate in real time the chlorination status of different water points in the city’s water supply system. Representatives from nine WASH Cluster partner organizations assembled into 13 groups of 1–3 persons to test water points in the 20 kebeles, administrative divisions, of Jijiga. Each group tested water samples from two water trucks, five tapstands, five donkey carts, three birkats (underground storage tanks), and five household containers. However, if a certain type of water point, for example water trucks, were unavailable in their assigned area, they were advised to test alternate water points. The number of samples determined to be taken from the piped system followed recommended WHO standards for sampling piped drinking water systems (WHO 1997). Each group was instructed to take samples from different geographic areas of their assigned kebeles in order to ensure that different areas were assessed. Household jerry cans were tested to see if chlorinated water was present at the household level after collection from points targeted with centralized or decentralized chlorination methods.

Each partner organization provided their own transportation, and chlorine test kits were provided to the organization for the assessment. FRC was tested using the *N*,*N*-diethyl-phenylenediamine (DPD) colorimetric method with Hach CN66 test kits (Hach Co., Loveland, CO) or Palintest pool testers (Palintest^®^, UK). All assessment data collection and entry were conducted on Samsung phones, using the free and open-source software Open Data Kit (ODK) software version 1.4. Paper forms were used when phones were unavailable. Verbal consent was obtained from the head of household or person responsible for delivering water before conducting water testing. The Somali RWB granted permission for the rapid assessment. Results are reported descriptively by water point type. Spatial results were produced using Quantum GIS (QGIS) software version 2.18.

## RESULTS

Overall, groups visited and tested 179 water points in Jijiga during the FRC rapid assessment: 36 tapstands from the piped network, 7 tapstands from boreholes, 47 donkey carts, 3 trucks that filled at trucking filling stations, 4 trucks that filled from surface water sources, 11 underground storage tanks connected to the piped network, 10 underground storage tanks filled with runoff water, and 61 household jerry cans. Tapstands connected to individual boreholes, underground storage tanks, and trucks that filled from surface water sources were not targeted by chlorination interventions, due to a lack of feasibility. In addition, data from two kebeles could not be collected due to time restrictions.

**Figure 1 ∣:**
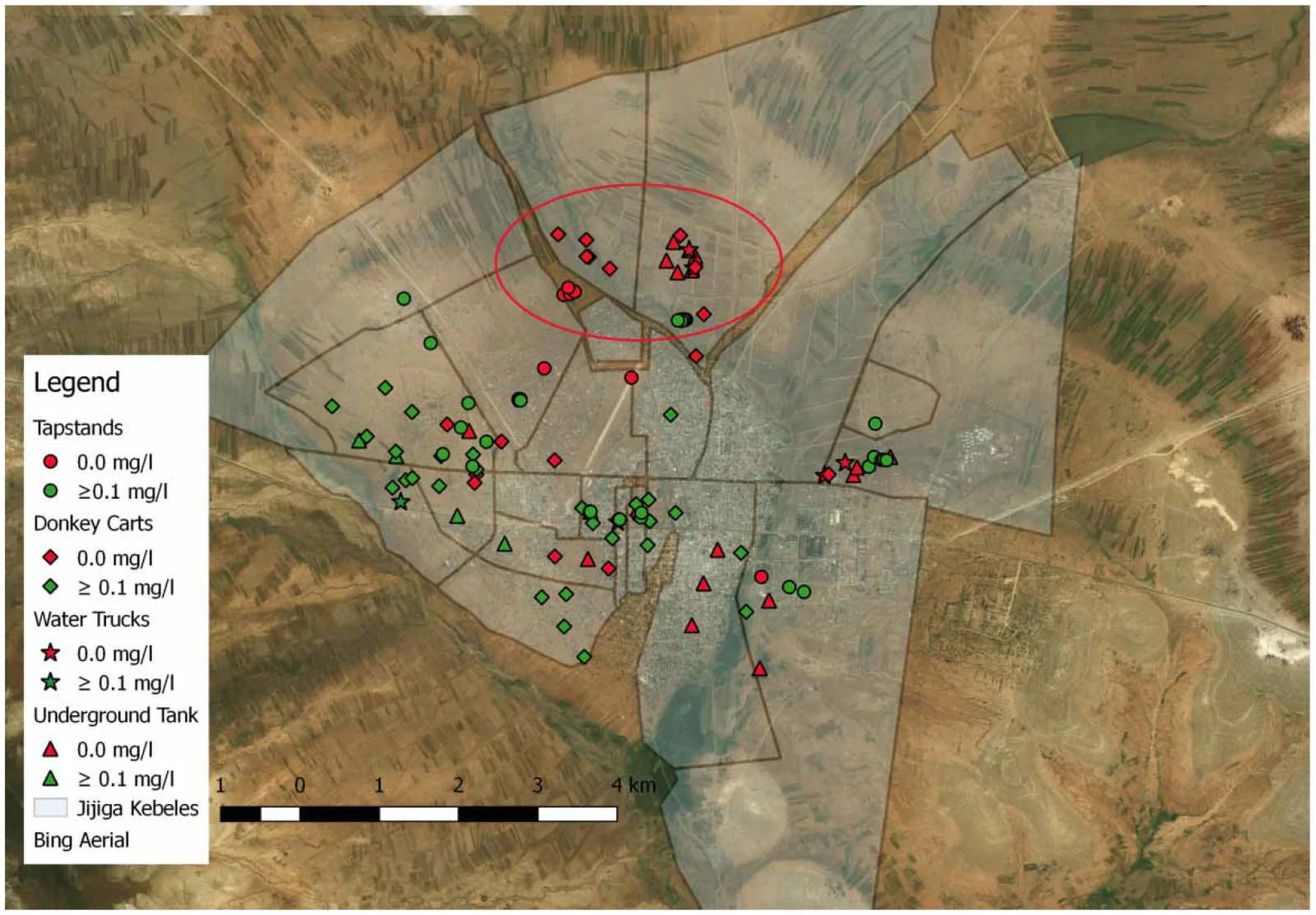
Spatial distribution of FRC data collected from tapstands, donkey carts, water trucks, and underground storage tanks during the rapid assessment in Jijiga, Somali Region. The figure also includes points not targeted by centralized and decentralized chlorination efforts, including tapstands connected to boreholes and water trucks and underground storage tanks filled with surface water. This spatial information was used to find five additional donkey cart filling stations in the north of Jijiga within the red circle. Decentralized chlorination was implemented at these points.

**Figure 2 ∣:**
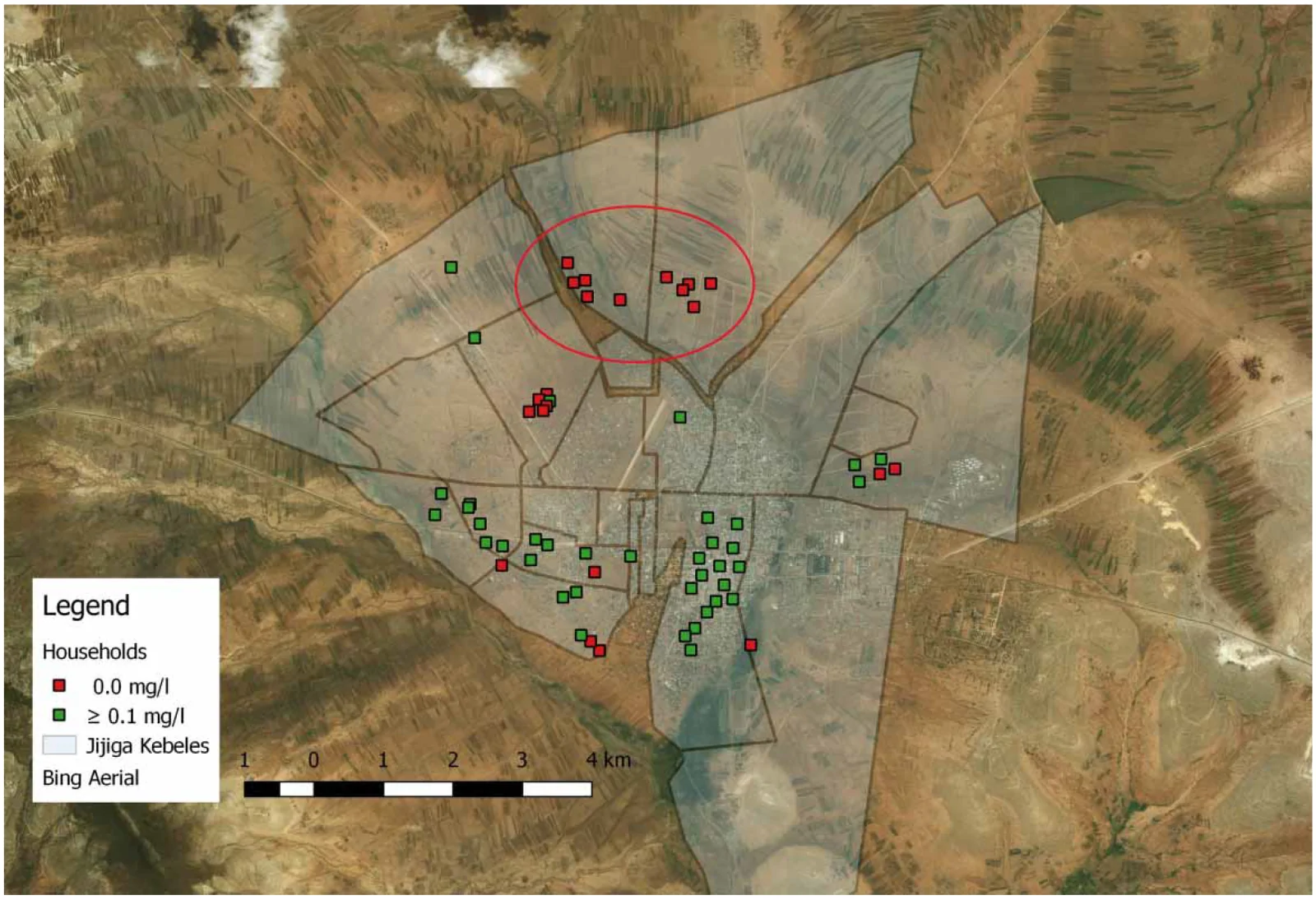
Spatial distribution of FRC data collected from household jerry cans during the rapid assessment in Jijiga, Somali Region.

In summary, 86% (31/36) of tapstands from the piped network, 60% (28/47) of donkey carts, 100% (3/3) of trucks, and 45% (5/11) underground storage tanks from the piped network tested had detectable FRC. In addition, 62% (38/61) of samples from household jerry cans had detectable FRC. These results are shown in Table 1. The spatial distribution of results is shown in Figures 1 and 2. Data collected using paper forms did not collect GPS points; thus, three data points are missing GPS locations.

## DISCUSSION

Approximately 1 month after chlorination activities started, we were able to detect FRC at the majority of water points tested and targeted with centralized or decentralized chlorination interventions. In addition, we also detected FRC in the majority of household water storage containers tested in various areas of Jijiga. This improvement in chlorination appeared to coincide with a stabilization of the incidence of AWD cases, although there is no clear evidence that this was directly attributable to the intervention. The implementation of chlorination interventions was guided by surveillance and case investigation data. Data from suspect AWD cases indicated that specific locations within the city were affected. Case investigations were conducted at the household level and included WASH questions such as water source. Data collected from these investigations allowed for the identification of gaps within the water supply system in Jijiga. Identification of these gaps led to further investigation of the water supply infrastructure and development of this community-level water quality intervention and monitoring system that targeted both the piped network and filling stations. This collaborative effort highlights the importance of a well-coordinated Health and WASH response to waterborne disease outbreaks.

Recent systematic reviews of WASH response during cholera outbreaks and emergencies highlighted that water quality interventions implemented during outbreaks and emergencies have not been well-documented (Ramesh et al. 2015; Taylor et al. 2015; Branz et al. 2017). In addition, community-level chlorination interventions implemented during outbreaks are not well documented in the literature apart from well chlorination programs (Garandeau et al. 2006; Guevart et al. 2008; Cavallaro et al. 2011; Branz et al. 2017). Our work demonstrates that water quality improvements can be implemented and documented quickly during an outbreak. Moreover, the FRC rapid assessment described in this report allowed for the timely collection of water quality data that resulted in targeted action in the north of Jijiga where several water points were not chlorinated. After this assessment, we identified five previously unknown donkey cart filling stations servicing these areas. Staff at these stations were subsequently trained and provided supplies for chlorination. This rapid assessment was successful in documenting improvements in water quality and identifying gaps in treatment. For sustained impact, periodic water quality monitoring will be essential to maintain consistent chlorination levels.

While free chlorine was detected in the majority of truck and donkey cart water tested, only a small proportion of these levels were >1.0 mg/L, the recommended level. There are two potential explanations for this. First, trucks and donkey carts that filled from filling stations often had long transport times before reaching delivery points. Thus, FRC levels likely decreased over time, but remained detectable. Second, donkey carts and truck tanks were not flushed out regularly, and thus, tanks could have organic matter inside which would increase the chlorine demand of the water inside the tanks. Longer transportation times after treatment, high chlorine demand due to organic matter, and higher temperature are all factors that have been documented in the literature to contribute to residual chlorine decay (CDC 2008; Ali et al. 2015; Nouri et al. 2015).

The distribution of household water treatment products is common during outbreaks and has been shown to improve the microbiological quality of water when paired with appropriate training and follow-up by community health workers, to ensure that households understand the methods of treatment and when to use products (Ramesh et al. 2015; Branz et al. 2017; Lantagne & Yates 2018). However, it can take time for water treatment behavior change to occur at the household level and monitoring visits are often used to reinforce messaging, as households may choose to not use the tablets or use the incorrect dose (Clasen 2009; Fiebelkorn et al. 20I2). During this outbreak response, centralized chlorination of the pipe network and decentralized chlorination at filling stations were prioritized. The advantage of this approach was that chlorinated water could be provided to a larger population in a shorter amount of time. In addition, this approach allowed for monitoring visits to be simplified and required visits to fewer key locations like filling stations and tapstands in certain areas. Centralized and decentralized chlorination methods could not be implemented in all areas of Jijiga and these areas were targeted with household water treatment methods. However, efforts should be made to avoid providing households collecting water from chlorinated water sources with household water treatment products, to prevent double dosing, as the resultant higher level of chlorine in drinking water may lead to rejection due to stronger chlorine taste and smell (Lantagne 2008; Branz et al. 2017).

The work in Jijiga is an example of a rapid targeted WASH intervention and assessment to respond to an ongoing outbreak and prevent further cases of AWD. This targeted response and assessment highlights that during an outbreak, community-level chlorination improvements can be rapidly implemented and assessed to ensure treated water is reaching affected communities.

## Figures and Tables

**Table 1 ∣:** FRC results from all water points tested

Sampling point	Total number of samples (*N*)	Samples with detectable FRC (*n*)	Samples with FRC between 0.1–0.5 mg/L (*n*)	Samples with FRC between 0.6–1.0 mg/L (*n*)	Samples with FRC >1.0 mg/L (*n*)
**Water points targeted with centralized and decentralized chlorination**					
Tapstand – piped network	36	86% (31)	47% (17)	36% (13)	3% (1)
Donkey cart	47	60% (28)	47% (22)	13% (6)	0% (0)
Truck – filling station	3	100% (3)	67% (2)	33% (1)	0% (0)
Underground tank (Birkat) – piped network*	11	45% (5)	36% (4)	9% (1)	0% (0)
*Total*	97	69% (67)	46% (45)	22% (21)	1% (1)
**Other water points not targeted by chlorination efforts**					
Tapstand – borehole	7	0% (0)	0% (0)	0% (0)	0% (0)
Truck – surface water	4	0% (0)	0% (0)	0% (0)	0% (0)
Underground tank (Birkat) – surface water**	10	0% (0)	0% (0)	0% (0)	0% (0)
*Total*	21	0% (0)	0% (0)	0% (0)	0% (0)
**Household level**					
Household Jerry can	61	62% (38)	43% (26)	16% (10)	3% (2)

* Underground tank filled with piped network water.

** Underground tank filled with surface water .
